# Prophylactic IABP Use in Protected PCI Reduces Infarction Size and Leads to More Complete Revascularization in Comparison to Rescue IAPB Use

**DOI:** 10.1002/ccd.31653

**Published:** 2025-06-02

**Authors:** Sascha d'Almeida, Tilman Stephan, Sebastian Weinig, Dominik Felbel, Benjamin Mayer, Stefanie Andreß, Wolfgang Rottbauer, Dominik Buckert, Sinisa Markovic

**Affiliations:** ^1^ Department of Cardiology, Angiology, Pneumology and Intensive Care Medicine University of Ulm Ulm Germany; ^2^ Institute for Epidemiology and Medical Biometry Ulm University Ulm Germany

**Keywords:** CAD, myocardial perfusion, prophylactic IABP, protected PCI, rescue IABP

## Abstract

**Background:**

There is little data questioning the timing of intra‐aortic balloon pump (IABP) implantation in non‐cardiogenic shock patients undergoing high‐risk percutaneous procedures.

**Aims:**

We compared prophylactic IABP (P‐IABP) implantation to an emergent, unplanned rescue use (R‐IABP) in high‐risk PCI.

**Methods:**

Among 300 IAPB patients who were treated at Ulm University Heart Center, Germany, between 2012 and 2020, we retrospectively selected and analyzed data from 59 patients. The cohort was subdivided into 44 P‐IABP and 15 R‐IAPB patients who underwent protected PCI with an IABP. Patients with cardiogenic shock at baseline, Impella‐pump or extra corporal membrane oxygenator (ECMO) were excluded. Both elective and emergency patients with acute coronary syndrome were included.

**Results:**

Both groups showed no significant difference in the baseline characteristics. The achieved SYNTAX score reduction after PCI (delta SYNTAX) was higher in the P‐IABP group (22.15 ± 10.31 points in the P‐IAPB and 15.73 ± 10.13 points in the R‐IABP group, *p* = 0.04). In addition, we observed lower highly sensitive Troponin T (hsTnT) peak values in the P‐IAPB group after the intervention (2223.33 ± 3129.77 ng/L vs. 5823.85 ± 3885.35 ng/L, *p* = 0.001). P‐IABP was associated with peak hsTnT values (*p* = 0.01). The 30‐day mortality rates were not significantly different (*p* = 0.88).

**Conclusion:**

Patients in the prophylactic‐IAPB group experienced a more complete revascularization measured with the delta SYNTAX score compared to those in the rescue‐IAPB group. Moreover, peri‐interventional infarct size measured by hsTnT release was significantly lower. Both findings indicate that P‐IABP implantation in high‐risk PCI should be preferred to rescue IAPB use.

## 1 Introduction

Amongst mechanical microcirculatory support devices, the Intra‐aortic balloon counter pulsation pump (IABP) is a widely established tool to stabilize selected patients with impaired left ventricular function undergoing cardiac procedures, that is, protected PCI or Mitraclip [1, 2, 3], especially in cases where a significant and rapid decrease of cardiac function is to be expected. There is sufficient data supporting the indication for mechanical support by IABP or Impella during high‐risk percutaneous intervention and contributing to attenuate the risk factors defining high‐risk PCI. However, recent trials investigating the effect of IABP support during PCI in patients with compromised cardiac or circulatory status, such as CRISP‐AMI [4] and SHOCK II [5], have not shown a reduction in infarct size or improvement in survival rates [4, 5]. This has led to a widespread restriction of IABP use in protected PCI. Nowadays, current guidelines limit the recommendation for prophylactic IABP (P‐IABP) implantation to cases of expected complex PCI in high‐risk patients, while in cases with only one of these criteria, it is recommended to reserve IABP as back‐up [6, 7]. The PROTECT II study, that compares the use of IABP and Impella in planned high‐risk PCI [8], shows comparable results with a trend toward a better outcome with Impella. Importantly, for Impella, there is data describing the ideal timing, advocating for an early implantation, if necessary [5]. However, there is very little data analyzing the timing of IABP implantation in stable and elective or acute coronary syndrome patients outside cardiogenic shock and undergoing high‐risk percutaneous procedures, which can objectively guide clinicians in decision making.

Thus, the present study compared the outcomes of non‐cardiogenic shock patients undergoing high‐risk PCI with early, P‐IABP and later, peri‐interventional, rescue IABP (R‐IABP) implantation and sought for predictors of R‐IABP implantation.

## 2 Methods and Study Population

We retrospectively analyzed data from 300 patients who underwent a percutaneous coronary intervention (PCI) with IAPB support at our University Heart Center in Ulm, Germany between 2012 and 2020. Among the total cohort, we finally included 59 patients, and classified them according to the time of insertion in a rescue (or bailout) IABP group (R‐IAPB), or P‐IABP group (P‐IAPB). While the majority of 44 patients received a P‐IAPB, 15 patients needed an unplanned R‐IAPB. Within the cohort, the only procedure used that could add complexity to our analysis was rotablation in complete total occlusion (CTO). In most patients with multivessel CAD, a staged procedure was preferred.

**Figure 1:**
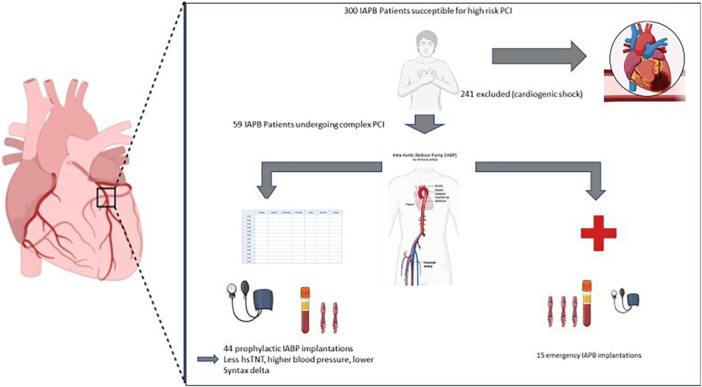
Flowchart of the analysis (created with Biorender).

The majority of patients (241 patients) were excluded as they were defined as non‐hemodynamically stable, presenting with or without acute myocardial infarction and systolic blood pressure under 90 mmHg or low output failure requiring catecholamines. We did not differentiate between the use of epinephrine, norepinephrine, or dobutamine. Although some patients with low catecholamines were not in shock or only in imminent cardiogenic shock, we tried to homogenize the data for comparability and excluded all patients with catecholamines. Patients who received other hemodynamic support devices were also excluded. Patients in shock were excluded, based on the results of the late IABP‐SHOCK II Study [5]. Patients were categorized in the R‐IAPB due to unexpected worsening of periprocedural circulatory situation; the rest of the patients received the elective IABP due to discretion of the interventionalist and were categorized as P‐IAPB. A follow‐up of 30 days and 6 months after the index procedure was part of the clinical routine and evaluated for study purposes as well. All relevant data was gathered from our SAP‐based hospital information system. The study design is displayed in Figure 1.

Baseline parameters, including demographics, comorbidities, patients cardiac history, medications, 12‐lead ECG, electrocardiographic, coronary angiographic, and laboratory parameters, were assessed at admission, respectively at the index left heart catheterization. Procedural parameters included data on the procedure performed and peri‐interventional complications. Outcome parameters, including echocardiographic and laboratory parameters, were assessed during the hospital stay, and patients were followed up for clinical events afterwards.

The study was approved by the local ethics committee (Ethics Application No. 40.19) and adheres to the Declaration of Helsinki.

Left ventricular function was assessed by levocardiography performed the index procedure and recorded in two projections (30 RAO/0 cranial and 40 LAO/0 cranial) routinely and classified into four categories: preserved/normal (1), slightly reduced (2), moderately reduced (3), and highly reduced (4) according to guideline‐specific recommendations [9]. After the intervention, a routine evaluation of the left ventricular function was done by echocardiography (LVEF). Laboratory parameters, including high‐sensitivity troponin T (hsTnT), were measured from blood samples.

The main variables of interest were the 30‐day and 6‐month survival rates after the index procedure. In addition, we created a combined endpoint of death, unplanned revascularization, cardiac hospitalization (i.e., decongestion), and spontaneous myocardial infarction within 6 months. Secondary endpoints were procedure‐related complications and post‐interventional parameters of hemodynamic and cardiac status, including residual Syntax score, hsTnT, and LVEF.

For continuous variables, we used the mean value ± SD, and we compared them with a *t*‐test or Mann–Whitney test for unpaired comparisons. Categorical variables were presented as counts and percentages and were compared by *χ*² (chi‐square) test. Differences were considered statistically significant when *p* < 0.05.

Both groups were compared in terms of baseline and periprocedural characteristics, assuming that statistically different variables between groups could give a hint to the best timing for IABP use. Point‐biserial correlation was performed to examine the effect of P‐IABP on peak hsTnT values. Univariate binary logistic regression analysis, followed by a multivariate logistic regression analysis by forward likelihood, with significant predictors from univariate analysis, was performed to uncover independent variables. Multivariate Cox regression analysis was performed to assess the association between male sex and the risk of in‐hospital mortality while adjusting for potentially confounding baseline characteristics. ST‐segment elevation was included as the primary independent variable. Baseline characteristics with a *p* < 0.05 in the univariate comparisons between the groups were included in the multivariate analysis. The enter method was applied for variable inclusion in the multivariate Cox regression analysis, and the proportional hazards assumption was tested by creating interaction terms, with violations addressed by stratification or exclusion of problematic variables. The strength of the association was expressed as a hazard ratio (HR) with 95%‐confidence interval (CI). The effect size *f* ^2^ was calculated based on Nagelkerke's *R* ^2^. According to Cohen's definition, an *f* ^2^ of at least 0.02 was considered small, 0.15 medium, and 0.35 large. *F*‐test was then used to assess the required sample size and the power of the model, employing G*Power for the estimation. A minimum power threshold of 0.8 was set to indicate sufficient statistical power. Statistical analysis was performed mainly with SPSS, version 20.0 (SPSS Inc., Chicago, Illinois & IBM in New York) and Python, version 3.8.

## 3 Results

Patients in the P‐IABP group were on average 74.02 ± 9.56 years old and 70.93 ± 12.57 in the R‐IAPB group (*p* = 0.33). Both groups were mainly male patients (67.4% in the P‐IABP and 80% in the R‐IAPB group, *p* = 0.36). Emergent admissions to the hospital occurred in 76.7% of the cases in the P‐IAPB group, compared to 100% in the R‐IAPB group (*p* = 0.04). On average, in both groups left ventricular ejection fraction was moderately or highly reduced, with a score between 3 and 4, (*p* = 0.43). Procedural length, periprocedural complications, and need for periprocedural reanimation or defibrillation were comparable in both groups.

Within the P‐IAPB group, one patient developed cardiogenic shock during the procedure. No cardiogenic shock occurred in the R‐IAPB group.

The main vessel of primary intervention, when allocatable, was the left main for the P‐IAPB group (56%) and the LAD (38%) for the R‐IABP group. The left main was the coronary of main intervention in 31% of cases in the R‐IAPB group (*p* = 0.43). In said vessel, the mean TIMI flow was 1.37 ± 0.75 in the p‐IAPB group and 1.28 ± 0.88 in the R‐IAPB group (*p* = 0.66). On average, 3.02 ± 1.76 stents were placed in the P‐IABP group and 2.47 ± 1.45 stents were placed (*p* = 0.26) in the R‐IAPB group. The procedure time this was performed in was not significantly different with 1.54 ± 0.65 h in the p‐IABP group and 1.49 ± 0.6 h in the R‐IAPB group (*p* = 0.78).

The syntax score before the intervention was 31.65 ± 8.94 points in the P‐IABP group and 31.33 ± 8.95 points in the R‐IAPB group (*p* = 0.91). The residual syntax score was 9.5 ± 10.12 points in the P‐IAPB and 15.60 ± 13.79 points in the R‐IAPB group (*p* = 0.13). The difference between both syntax scores, the syntax‐delta as a measure of the success of revascularization was 22.15 ± 10.31 points for the IAPB group and 15.73 ± 10.13 points for the R‐IAPB group (*p* = 0.04).

While aortic stenosis was equally distributed between both groups, affecting around 8% of both collectives (*p* = 0.96), rates of severe mitral regurgitation were twice as high in the P‐IAPB group, not reaching the significance value (*p* = 0.52). Moreover, patients were more likely to receive a P‐IABP when they were endangered by a history of atrial flutter (*p* = 0.05) or a left main stenosis (*p* = 0.06). Baseline values for both systolic and diastolic blood pressure, heart rate, LVEDP, and sPAP, history of cardiac surgery or intervention did not seem to influence the time of IABP implantation. R‐IAPB patients more often displayed ST Segment elevation (*p* = 0.01). Main LVEF after PCI was reduced in both groups, with a slightly better mean value of 39.41 ± 17.19% in the P‐IAPB group and 34.91 ± 12.10 in the R‐IAPB group (*p* = 0.44). In the P‐IAPB group, the post‐interventional systolic blood pressure was significantly higher than in the R‐IAPB group (131.97 ± 22.80 mmHg vs. 108.75 ± 10.38 mmHg, *p* = 0.02). Intake of all medication influencing blood pressure was no different (*p* > 0.16). The equivalent diastolic blood pressure did not differ either (*p* = 0.69).

While hsTnT levels before PCI were higher in the P‐IABP group (738.76 ± 1339.61 ng/L vs. 423.0 ± 787.22 ng/L, *p* = 0.46). HsTnT levels spiked significantly higher in R‐IAPB group, (5823.85 ± 3885.55 ng/L, compared to 2223.33 ± 3129.77 ng/L, *p* = 0.001). Point‐biserial correlation demonstrated that P‐IABP was associated with lower peak hsTnT values (*p* = 0.01).

Thirty‐day mortality was comparable in both cohorts (15.0% vs. 13.3%, *p* = 0.88). Six‐month‐mortality was 18.4% in the P‐IABP group and 36.3% in the R‐IAPB group (*p* = 0.24).

All baseline characteristics are displayed in Table 1, procedural data in Table 2, and outcome data in Table 3.

**Table 1:** Baseline characteristics: Comparison of P‐IABP and R‐IABP‐backed patients.

Baseline parameters (at admission)	P‐IABP	R‐IABP	*p* value
Baseline characteristics			
*N*, number of patients	44	15	
Age (years)	74.02 ± 9.56	70.93 ± 12.57	0.33
Male sex *n*, (%)	29 (67.4)	12 (80)	0.36
Emergency referral *n*, (%)	34 (76.7)	15 (100)	**0.04**
Elective referral *n*, (%)	10 (23.2)	0 (0.0)	**0.04**
Cardiological baseline parameters			
LVEF‐score in laevocardiography	3.44 ± 1.01	3.73 ± 0.46	0.43
Preserved LVEF (1) *n*, (%)	5 (11.3%)	0 (0%)	
Slightly reduced LVEF (2) *n*, (%)	1 (2.3%)	0 (0%)	
Moderately reduced LVEF (3) *n*, (%)	7 (15.9%)	4 (26.7%)	
Highly reduced LVEF (4) *n*, (%)	31 (70.5%)	11 (73.3%)	
Syntax‐score	31.65 ± 8.94	31.33 ± 8.95	0.91
Unprotected left main stenosis *n*, (%)	31 (73.8)	7 (46.7)	0.06
Proximal RCA stenosis *n*, (%)	21 (51.2)	7 (46.7)	0.76
Aortic stenosis *n*, (%)	3 (7.9)	1 (8.3)	0.96
Mitral insufficiency *n*, (%)	6 (15.8)	1 (8.3)	0.52
History of atrial flutter *n*, (%)	9 (20.9)	0 (0.0)	0.05
History of stent implantation *n*, (%)	11 (44.0)	3 (30.0)	0.45
History of bypass surgery *n*, (%)	2 (4.7)	2 (13.3)	0.25
Systolic pulmonal arterial pressure, sPAP (mmHg)	52.61 ± 14.87	57.80 ± 12.32	0.48
Aortal diameter (mm)	31.32 ± 4.93	29.40 ± 5.08	0.29
Heart rate before the procedure (min^−1^)	83.15 ± 20.15	83.71 ± 17.70	0.93
Systolic blood pressure before the procedure (mmHg)	131.97 ± 22.80	125.55 ± 22.48	0.42
Diastolic blood pressure before the procedure (mmHg)	71.28 ± 11.46	70.82 ± 12.24	0.91
ECG‐parameters			
Sinus rhythm *n*, (%)	38 (88.4)	15 (100)	0.17
Atrial flutter *n*, (%)	4 (9.3)	0 (0.0)	0.22
Left bundle branch block *n*, (%)	9 (21.4)	3 (20.0)	0.91
Left anterior branch block *n*, (%)	1 (25.0)	3 (20.0)	**0.02**
Right bundle branch block *n*, (%)	5 (12.2)	4 (30.8)	0.12
ST‐segment elevation *n*, (%)	10 (23.3)	9 (60.0)	**0.01**
ST segment depression *n*, (%)	13 (31.0)	8 (53.3)	0.12
T‐wave inversion *n*, (%)	38 (90.5)	12 (80.0)	0.29
Lab values			
Lactate dehydrogenase (LDH) in U/L	317.23 ± 162.01	385.18 ± 269.08	0.33
NT‐proBNP (pg/mL)	11836 ± 11279	10443 ± 11688	0.75
GFR (mL/min)	56.5 ± 25.07	53 ± 22.52	0.67
Creatinine (µmol/L)	126.97 ± 72.75	127.62 ± 60.79	0.98
Bicarbonate levels (mmol/L)	23.37 ± 4.15	21.81 ± 3.06	0.27
Base Excess in (mmol/L)	−1.05 ± 3.70	−3.65 ± 3.91	0.06
Creatinine kinase in (U/L)	431.81 ± 849.13	258.67 ± 404.04	0.56
(hsTnT) before PCI in (ng/L)	738.76 ± 1339.61	423.0 ± 787.22	0.46
Medication			
ACE‐inhibitors *n*, (%)	22 (52.4)	11 (73.3)	0.16
ß‐blocker *n*, (%)	40 (95.2)	15 (100)	0.39
AT1‐antagonist *n*, (%)	15 (35.7)	3 (20.0)	0.26

*Note:* Bold values are considered significant since their *p* value is lower than 0.05. Total (*n*) may differ for certain parameters, as they could not always be assessed for every patient retrospectively. *p* values were determined by Student's *t*‐test and *χ*² test.

**Table 2:** Procedural data: Comparison of P‐IABP and R‐IABP‐backed patients.

Procedural parameters	P‐IABP	R‐IABP	*p* value
*N*, number of patients	44	15	
Primary vessel of intervention	(*n* = 41)	(*n* = 13)	
LAD	10 (24.4%)	5 (38.4)	0.43
RCX	3 (7.3%)	2 (15.4%)	
RCA	5 (12.2%)	2 (15.4%)	
Left main	23 (56.1%)	4 (30.8%)	
Initial TIMI flow	1.37 ± 0.75	1.28 ± 0.88	0.66
Number of residual stenosed vessels^a^	1.22 ± 0.99	0.93 ± 0.95	0.32
Count of placed stents	3.02 ± 1.76	2.47 ± 1.45	0.26
Failed attempted lesion	3	2	0.43
Procedural Complications			
Intervention length in hours	1.54 ± 0.65	1.49 ± 0.60	0.78
Periprocedural CPR *n*, (%)	1 (2.3)	0 (0.0)	0.55
Periprocedural defibrillation *n*, (%)	3 (7.0)	2 (13.3)	0.45
Periprocedural cardiogenic shock *n*, (%)	1 (2.4)	0 (0.0)	0.55

*Note:* Bold values are considered significant since their *p* value is lower than 0.05. Total (*n*) may differ for certain parameters, as they could not always be assessed for every patient retrospectively. *p* values were determined by Student's *t*‐test and *χ*² test.

a After the index procedure.

**Table 3:** Outcome data with STEMI subgroup: Comparison of P‐IABP and R‐IABP‐backed patients.

Outcome parameters	P‐IABP	R‐IABP	*p* value
*N*, number of patients	44	15	
Cardiological outcome parameters			
Residual syntax‐score	9.5 ± 10.12	15.60 ± 13.79	0.13
Syntax‐score‐delta	22.15 ± 10.31	15.73 ± 10.13	**0.04**
LVEF after the intervention *n*, (%)	39.41 ± 17.19	34.91 ± 12.10	0.44
LVEDP after the procedure (mmHg)	28.69 ± 12.73	29.29 ± 9.05	0.86
Systolic blood pressure after the procedure (mmHg)	124.74 ± 22.07	108.75 ± 10.38	**0.02**
Diastolic blood pressure after the procedure (mmHg)	65.59 ± 9.59	64.25 ± 11.41	0.69
Lab values			
Creatinine (µmol/L)	126.97 ± 72.75	127.62 ± 60.79	0.98
Post‐procedural kidney injury^a^	8 (20.5%)	2 (18.2%)	0.68
Time elapsed until next hsTnT measurement (h)	3.68 ± 6.15	3.45 ± 4.63	0.89
HsTnT after PCI in (ng/L)^b^^,^ d	1408.3 ± 2395.1	4557 + 4137.8	0.001
Peak hsTnT value (ng/L)^d^	2223.33 ± 3129.77	5823.85 ± 3885.55	**0.001**
Follow‐up			
30‐day‐mortality *n*, (%)	6 (15.0)	2 (13.3)	0.88
6‐month‐mortality *n*, (%)	7 (18.4)	4 (36.3)	0.24
Adverse events^c^(6 months time)	8 (18.2%)	4 (26.3%)	0.48
STEMI subgroup analysis			
HsTnT after PCI^b^^,^ d	2970.0 ± 2914.0	6510.0 ± 3511.3	***p*** = **0.03**
HsTnT peak^d^	5574.4 ± 3510.9	6872.9 ± 3380.9	*p* = 0.4
Mortality (6 months)	3 (30%)	3 (33.3%)	*p* = 0.87

*Note:* Bold values are considered significant since their *p* value is lower than 0.05. Total (*n*) may differ for certain parameters, as they could not always be assessed for every patient retrospectively. *p* values were determined by Student's *t*‐test and *χ*² test.

a Chronic dialysis patients were excluded, *n* = 39 for P‐IABP and *n* = 11 for R‐IAPB group.

b Measured immediately after PCI.

c Adverse events were considered as the combined endpoint of death, unplanned revascularization, cardiac hospitalization (i.e., decongestion), and spontaneous myocardial infarction within 6 months.

^d^ Levels above 10,000 ng/L cannot be determined and are counted as 10,000 ng/L.

Multivariate regression analysis including all baseline characteristics that were significantly different in the univariate comparisons between the groups (covariates: emergency admission, left anterior branch block, history of myocardial infarction) showed that the only independent predictor for an R‐IABP implantation was ST segment elevations (*p *= 0.029, HR 4.551, 95% CI 1.165−17.782). The covariates included are shown in Supporting Information S1: Table 1.

Due to the rather small sample size, a post hoc power analysis was performed for the multivariate regression analysis. The calculated effect size was 0.524, demonstrating a large explanation of the effect by the model. Given the sample size of *n* = 59, the number of predictors of *k* = 4, and the significance level of *α* < 0.05, power analysis using G*Power showed that statistical power exceeded the set threshold of 0.8.

Within the subgroup with ST‐elevations, the hsTnT measured directly after the PCI was 2970.0 ± 2914.0 ng/L in the P‐IABP group and 6510.0 ± 3511.3 ng/L in the R‐IABP group (*p *= 0.03), while the peak hsTnT was 5574.4 ± 3510.9 ng/L in the P‐IAPB group and 6872.9 ± 3380.9 in the R‐IAPB group (*p *= 0.4) (Table 3).

## 4 Discussion

The aim of the study was to analyze the importance of the timing in IAPB implantation. In this retrospective analysis, we therefore compared P‐IABP implantation to an emergent, unplanned use (R‐IABP) in high‐risk PCI. The course of the study along with the main results are shown in Figure 1.

Our study reveals that a complex PCI with an early IAPB implantation leads to a significantly higher systolic postinterventional blood pressure and a lower maximum of post‐procedural hsTnT levels. Moreover, patients in the elective IABP (P‐IABP) cohort achieved a significant reduction of the initial syntax score, indicating a more complete revascularization. The 30‐day and 6‐month mortality rates were not significantly different. Albeit the 6‐month mortality in the rescue‐IAPB group was twice as high, it did not reach statistical significance.

It is important to emphasize that both groups were comparable. This is shown with comparable baseline parameters in both groups. The pre‐interventional syntax score, the initial blood pressure, the BNP levels, and the hsTnT levels before intervention as well as the intervention time, the initial lavocardiographic left ventricular ejection fraction, and the count of stents implanted were not significantly different. On the contrary, there is a tendency for more left main stenosis in the P‐IABP group. To contextualize the occurrence of more ST elevations in the R‐IABP group, we created a subgroup analysis only with STEMI patients. Here, hsTnT immediately after PCI was significantly lower in the R‐IAPB group in the STEMI subgroup. Peak hsTnT was not significant. This is partly due to the small number analyzed but also that lab values over 10,000 ng/L could not be narrowed down retrospectively.

Until now, especially the late Intra‐Aortic Balloon Pump II (IABP Shock II) Trial [5] has shaped the guidelines. Since that trial with 600 patients, the use of IABP has lost its importance in general and especially in patients presenting with cardiogenic shock. Patients in the said study reported a mortality of roughly 40% [5]. In addition, the therapy did not contribute to reducing the length of the stay in the intensive care facility [5]. Other trials like CRISP‐AMI showed no reduction of myocardial infarction size through P‐IAPB use [4]. In the CRISP‐AMI‐Study, not only did the control group not receive an IAPB, patients that had an indication for a P‐IAPB were explicitly excluded. As a result, even though the data of CRISP‐AMI shows that not every patient benefits from an IABP, it does not allow a conclusion about which patients should be treated with IAPB. In addition, it does not consider the timing or technical limitations of IABP. It is known for instance that effectiveness of IAPB decreases when systolic blood pressure drops under 60 mmHg or the heart rate rises above 140 min^−1^ [10] because of technical and cardiac [11] limitations.

In our study, patients presenting with cardiogenic shock were therefore excluded from the study. This means the R‐IAPB group was merely stabilized because of an imminent cardiocirculatory deterioration before any cardiogenic shock could take place. On the other hand, one P‐IAPB patient that underwent cardiogenic shock stayed in the study, since he underwent cardiogenic shock only after IAPB implantation. For comparison, in the SHOCK II study, IAPB implantation took place mostly (86.6%) after the PCI.

This makes the SHOCK II study (i.e., mostly based on R‐IAPB implantation) an unbalanced study regarding the timing of IABP implantation and emphasizes the results of our own study as it may put specific results of SHOCK II into perspective. Contrary to SHOCK II, in our trial, we analyzed a cohort that was mostly composed of P‐IAPB patients (75%). Moreover, in the SHOCK II Study, that claimed no effect in mortality, approximately 10% of patients that were designated to be in the non‐IAPB group were switched due to their cardiocirculatory worsening. In addition, 4.3% of patients in the IAPB group died before IAPB implantation. In essence, some of the IAPB limitations that arise from these aforementioned studies and consequently the indications for IAPB might need some further research.

Elective implantation of IABP before PCI was done at the interventionalist's discretion, and it is of course debatable to what extent each of the said variables have really led to increased IABP use in the P‐IABP group. Even with these independent parameters IAPB use remains a choice incumbent to the experience of the interventionalist and the expected complexity of the PCI. These subjective points limit the scope of the aforementioned data.

On the other hand, ST‐segment elevation ACS might have negatively influenced the choice for P‐IAPB use. Retrospectively, it is difficult to reenact the clinical decision that led to the use of a device. In cases with stable ST‐segment elevations, the necessity to intervene a patient as fast as possible might give a “time consuming IAPB implantation” a secondary priority, as “time is muscle.”

It is important to emphasize that the choice for P‐IABP always arises because of comorbidity that the patients has, while the rescue IAPB is mostly used because of unknown/unexpected comorbidities or acute worsening. Therefore, in theory, the P‐IAPB would attract more patients with a long list of known illnesses, such as renal dysfunction or pulmonary hypertonia that could undergo cardiac shock if not sufficiently IAPB backed. The R‐IABP would be for elective patients in which the severity of CAD was underestimated. In our cohort, the opposite seems to be the case. Significantly, more patients in the R‐IAPB group are emergencies. In fact, all R‐IAPB patients are emergencies, mostly because most elective patients' coronary status is known. Renal function and sPAP does not differ in both groups. In fact, in our cohort, ST segment elevations are the only statistically independent variable that might have influenced the choice for P‐IAPB. Regarding all the other variables including lab values, none influences the timing of IAPB implantation significantly.

Even though survival, measured after 30 days and 6 months, is not significantly influenced by the timing of the IAPB implantation in our study, other studies, especially Perera et al. [12], indicate a beneficial long‐term effect of the P‐IABP use. The data of Mishra et al. [13] that analyzed 114 patients with said question looks even more promising. P‐IAPB patients showed a lower mortality and a lower complication rate with an early IAPB implantation. It is quite arguable that the design of the aforementioned study by Mishra et al. and the number of patients that were consecutively recruited allow a broader statement about the effectiveness of an early IAPB implantation in terms of hard endpoints. However, our hereby presented data indicates confirms their promising results: Patients susceptible for elective IAPB in high‐risk PCI experience more frequently a complete revascularization with smaller infarction size as indicated by lower hsTnT levels [14]. The high mortality rates are consistent with previous studies. The CRISP‐AMI trial found a mortality rate of nearly 10% at 6 months in stable patients undergoing PCI for acute myocardial infarction [4], and an even higher mortality rate of approximately 40% at 30 days in patients with manifest cardiogenic shock [5]. These data show the poor prognosis of these patients, particularly as their circulatory condition deteriorates, and thus underscore the urgent need for effective treatment strategies in this cohort and the relevance of our findings.

In view of the relatively small sample size and the strong effect of the timing of IABP‐implantation on outcomes observed in our study, we set the conventional target statistical power of 0.8 for the multivariate analysis of predictors of R‐IABP implantation. As the model was highly performant with a large effect, and the power exceeded the set threshold of 0.8, inclusion of more patients was neither considered necessary nor justifiable.

In consideration of our results, the timing of IABP implantation appears to be critical for its effect in high‐risk PCI. The superiority of early P‐IABP support in our hemodynamically stable patient cohort is consistent with the lack of efficacy of IABP support in patients with severely impaired hemodynamic and cardiac status [4, 5, 8]. While CRISP‐AMI and SHOCK II, in contrast to our results, did not find a benefit of IABP support, it must be stated that these trials included patients with severely reduced LVEF [4], and manifest cardiogenic shock [5], respectively. This loss of IABP efficacy as patients' circulatory condition worsens may be due to the rather mild hemodynamic support provided by IABP, which is strengthened by the strong trend toward worse survival outcomes compared to Impella observed in the PROTECT II trial [7]. Overall, our results, in combination with the previous studies of CRISP‐AMI, SHOCK II, and PROTECT II trials, suggest a benefit of IABP implantation (only) in a stable, prophylactic setting and thus stress the importance of timing. These findings are consistent with the current European Society of Cardiology guidelines recommending P‐IABP implantation in high‐risk patients undergoing expected complex PCI. However, the diminishing effect of IABP with worsening of circulatory condition calls into question the recommendation that R‐IABP should be considered as a back‐up if only one of these criteria is present [6, 7].

Of course, the comparison of our results with other studies on IABP support during high‐risk PCI must be done with caution because of the differences in the cohorts studied so far. However, we want to highlight that the intervention of IABP implantation was similar. Thus, this different cohort of patients adds another comparison group for the effect of IABP implantation. Moreover, the lack of benefits of IABP support in patients with cardiogenic shock observed in SHOCK II [5] is consistent with the diminishing effect with worsening of circulatory condition observed in our study. Thus, we believe that this putative limiting factor may provide an explanation for the different results, and the comparison with SHOCK II is consistent with, extends, and strengthens our findings.

In literature, IAPB use is not unanimously accepted. Some studies claimed that IAPB could reduce coronary flow in certain cases [15]. This may also be linked to the fact, that IAPB effectiveness is dependent of various factors including blood pressure [16] but especially aortal compliance and vascular counter mechanisms less examined in literature [17, 18]. Nonetheless, most studies only focus on blood pressure as the main surrogate parameter. Exploring the role of other hemodynamic parameters could be useful in IABP research.

In the end, it is still up for discussion which patients might benefit from a protected PCI. Probably another randomized trial focusing on that question and focusing on the moment of IAPB implantation will bring definitive insight into the usefulness of the IABP.

This study has already been done with the Impella. Not only has an early implantation shown to be beneficial for patients [19]. Some data suggest that it might be beneficial in cardiogenic shock, unlike the present SHOCK II data with IAPB [20]. With our data suggesting benefits for IAPB in certain patients, we suggest that defining the right type of circulatory support in the right patient with the right timing might be the next challenge in device‐based treatment of heart failure (Central Illustration 1).

**Central Illustration 1:**
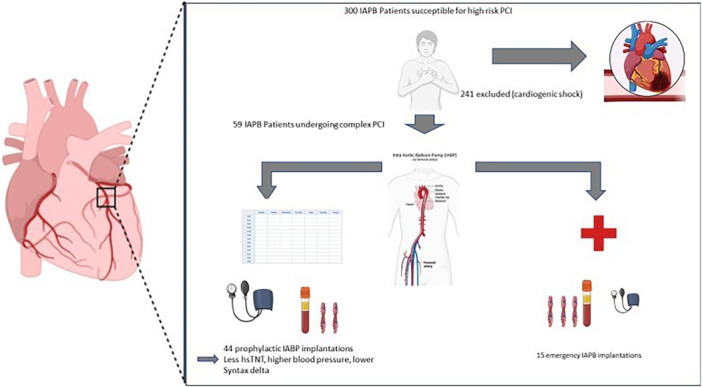
Within 300 patients susceptible for IAPB in high‐risk PCI, we retrospectively selected 59 patients without cardiogenic shock and showed that within this collective, prophylactic (P‐)IABP is associated with better revascularization and smaller infarct size than rescue (R‐)IABP.

## 5 Conclusion

While the usage in cardiogenic shock itself has been restricted by the latest studies, our study demonstrates that IABP still remains a common tool for circulatory support in the field of protected PCI. With only a few data on this subject, the aim of this retrospective study was to compare two cohorts: patients with P‐IABP implantation and emergent, unplanned (R‐IABP) implantation in stable patients both undergoing a high‐risk PCI.

While IAPB implantation always remains a case‐to‐case decision, we could show that patients in need of an IAPB may benefit from an early elective implantation. The study shows that an elective IABP implantation is associated with more frequently achieved complete revascularization (measured with the achieved delta SYNTAX score) and smaller infarct size as indicated by lower hsTnT levels, after percutaneous revascularization. Thus, this study again suggests that elective IABP implantation may have a possible clinical benefit for stable patients undergoing high‐risk percutaneous procedures. Moreover, our study suggests that patients with ST‐segment elevations are at risk of ending up with a R‐IABP implantation.

These findings indicate that recommendations regarding the use of IABP, especially regarding the timing of implantation, should be reevaluated and that further studies are needed in this field.

### 5.1 Limitations

We acknowledge that our IAPB groups are small cohorts with less than 100 patients, and the numbers differed between groups who were analyzed retrospectively. Therefore, the scope of our analysis is limited. However, due to the superiority of P‐IABP as shown by hsTnT levels and the far‐reaching consequences in this highly vulnerable cohort as demonstrated by the high mortality rates, we closed our study at this point. With a large effect size and the power of the multivariate analysis, the reliability of our results has been further strengthened. Moreover, the number of patients included differed between the groups. However, all patients who met the inclusion criteria in the given study period were considered to provide an unbiased insight. We also note that in the first place even with the presented independent parameters, no algorithm was followed to decide which patient should receive an IAPB. Its use remains a choice incumbent to the experience of the interventionalist and the expected complexity of the PCI. These subjective points limit the scope of the aforementioned data.

## Ethics Statement

The article has an approval from the Ethics committee of the University of Ulm. The approval is in accordance with the ethical standards laid down in the 1964 Declaration of Helsinki and its later amendments and well as with applicable German federal law.

## Conflicts of Interest

The authors declare no conflicts of interest.

## Supporting information

IABP supplementary material.
