# Supplementary material for: Prophylactic IABP Use in Protected PCI Reduces Infarction Size and Leads to More Complete Revascularization in Comparison to Rescue IAPB Use
Source: Catheter Cardiovasc Interv. 2025 Jun 2;106(2):1002–11. doi: 10.1002/ccd.31653 (PMC12336753; doi:10.1002/ccd.31653)
Supplement: Supplementary file 1 — IABP supplementary material. [file CCD-106-1002-s001.docx]

**Supplementary Material:**

| **Supplementary Table 1: Covariates in the multivariate analysis for rescue IABP-implantation including all baseline characteristics of significant difference in the univariate comparisons between the groups (p value < 0.05)** | | | |
| --- | --- | --- | --- |
|  | **p value** | **Hazard Ratio** | **95%-Confidence Interval** |
| Emergency admission | 0.999 | >10.000 | 0.000-n.c. |
| Left anterior branch block | 0.103 | 7.932 | 0.658-95.579 |
| History of Myocardial Infarction | 0.324 | 1.746 | 0.577-5.290 |
| IABP, Intra-Aortic Balloon Implantation;  n.c., not calculated | | | |
